# Surgical Standards for Management of the Axilla in Breast Cancer Clinical Trials with Pathological Complete Response Endpoint

**DOI:** 10.1038/s41523-018-0074-6

**Published:** 2018-08-17

**Authors:** Judy C. Boughey, Michael D. Alvarado, Rachael B. Lancaster, W. Fraser Symmans, Rita Mukhtar, Jasmine M. Wong, Cheryl A. Ewing, David A. Potter, Todd M. Tuttle, Tina J. Hieken, Jodi M. Carter, James W. Jakub, Henry G. Kaplan, Claire L. Buchanan, Nora T. Jaskowiak, Husain A. Sattar, Jeffrey Mueller, Rita Nanda, Claudine J. Isaacs, Paula R. Pohlmann, Filipa Lynce, Eleni A. Tousimis, Jay C. Zeck, M. Catherine Lee, Julie E. Lang, Paulette Mhawech-Fauceglia, Roshni Rao, Bret Taback, Constantine Godellas, Margaret Chen, Kevin M. Kalinsky, Hanina Hibshoosh, Brigid Killelea, Tara Sanft, Gillian L. Hirst, Smita Asare, Jeffrey B. Matthews, Jane Perlmutter, Laura J. Esserman, and I-SPY 2 Investigators, A. Jo Chien, Andres Forero-Torres, Douglas Yee, Erin D. Ellis, Heather S. Han, Janice Lu, Anne M. Wallace, Kathy S. Albain, Anthony D. Elias, Amy S. Clark, Kathleen Kemmer

**Affiliations:** 1 Department of Surgery, Mayo Clinic, Rochester, MN USA; 2 UCSF Heller Diller Family Comprehensive Cancer Center, San Francisco, CA USA; 3 Department of Surgery, University of Alabama at Birmingham, Birmingham, AL USA; 4 Department of Pathology, MD Anderson Cancer Center, Houston, TX USA; 5 Department of Medicine, University of Minnesota, Minneapolis, MN USA; 6 Department of Surgery, University of Minnesota, Minneapolis, MN USA; 7 Department of Pathology, Mayo Clinic, Rochester, MN USA; 8 Swedish, Swedish Cancer Institute, Seattle, WA USA; 9 Department of Surgery, University of Chicago, Chicago, IL USA; 10 Department of Pathology, University of Chicago, Chicago, IL USA; 11 Department of Hematology and Oncology, University of Chicago, Chicago, IL USA; 12 Georgetown University Medical Center, Lombardi Cancer Center, Washington, DC USA; 13 Moffitt Cancer Center, Tampa, FL USA; 14 University of Southern California, Norris Comprehensive Cancer Center, Los Angeles, CA USA; 15 Columbia University Medical Center, New York, NY USA; 16 Loyola University Medical Centre, Maywood, IL USA; 17 Department of Surgery and Department of Medical Oncology, Yale University, New Haven, CT USA; 18 Quantum Leap Health Care Collaborative, San Francisco, CA USA; 19 Patient Representative, Ann Arbor, Michigan USA; 20 Department of Breast Oncology, Moffitt Cancer Center, Tampa, FL USA; 21 University of California, San Diego, La Jolla, CA USA; 22 University of Colorado Denver, Denver, CO USA; 23 University of Pennsylvania, Philadelphia, PA USA; 24 Oregon Health and Sciences, Portland, OR USA

## Abstract

Advances in the surgical management of the axilla in patients treated with neoadjuvant chemotherapy, especially those with node positive disease at diagnosis, have led to changes in practice and more judicious use of axillary lymph node dissection that may minimize morbidity from surgery. However, there is still significant confusion about how to optimally manage the axilla, resulting in variation among practices. From the viewpoint of drug development, assessment of response to neoadjuvant chemotherapy remains paramount and appropriate assessment of residual disease—the primary endpoint of many drug therapy trials in the neoadjuvant setting—is critical. Therefore decreasing the variability, especially in a multicenter clinical trial setting, and establishing a minimum standard to ensure consistency in clinical trial data, without mandating axillary lymph node dissection, for all patients is necessary. The key elements which include proper staging and identification of nodal involvement at diagnosis, and appropriately targeted management of the axilla at the time of surgical resection are presented. The following protocols have been adopted as standard procedure by the I-SPY2 trial for management of axilla in patients with node positive disease, and present a framework for prospective clinical trials and practice.

## Introduction

The use of neoadjuvant systemic treatment in the clinical care of patients with breast cancer has increased dramatically over recent years. For patients with breast cancer with high-risk biologic features, in particular triple negative breast cancer (TNBC) and HER2-positive breast cancer, it is becoming the primary recommendation. The neoadjuvant setting also offers important advantages that have enabled accelerated development of new drugs for women with breast cancer. In 2012, the US Food and Drug Administration (FDA) released guidance indicating they would consider granting accelerated drug approval on the basis of a surrogate endpoint that is reasonably likely to predict clinical benefit. For neoadjuvant breast cancer treatment they proposed the rate of pathological complete response (pCR)—the complete eradication of disease—as a surrogate.^1^ This validated and highlighted the importance of the neoadjuvant setting for assessment of therapeutic response in breast cancer. As clinical trials involving neoadjuvant systemic therapy which include pathologic complete response as the primary endpoint increase, standardizing the surgical management in these trials is important in the face of evolving surgical techniques, in particular as it relates to the management of the axilla. Herein, we present surgical standards for management of the axilla in patients treated with neoadjuvant chemotherapy on clinical trials.

The definition of pCR varies across different clinical trials. Of the three most common definitions of pCR—ypT0/is, ypT0/is ypN0, ypT0 ypN0—those that best correlate with survival incorporate assessment of both the breast and the lymph nodes (ypT0/is ypN0, and ypT0 ypN0).^2^ Therefore, for clinical trials of novel agents in which pCR is the primary endpoint, these definitions—the absence of tumor in the breast as well as in the axillary lymph nodes—are the preferred definitions, reinforcing the importance of accurate assessment of axillary nodes for disease after neoadjuvant chemotherapy.

One significant advance in the surgical management of patients treated with neoadjuvant chemotherapy is the potential to allow less invasive surgical intervention in the axilla for those patients who have an excellent response to neoadjuvant chemotherapy. Staging of axillary lymph nodes has generally been performed by sentinel lymph node (SLN) surgery. However, SLN surgery in all settings is associated with a false negative rate (FNR). SLN in the absence of neoadjuvant therapy is associated with a FNR of 6–11%^3–6^; in the setting of node-negative disease treated with neoadjuvant chemotherapy, surgical staging of the axilla after NAC by SLN has a similar FNR of 6–12%.^7–11^ Historically, in node positive disease, ALND was recommended to ensure resection of all nodes in level I and II of the axilla for pathologic evaluation. However, there is now evidence from prospective clinical trials to support the use of SLN surgery after neoadjuvant chemotherapy for patients presenting with clinically node positive disease who have a good clinical and imaging response.^12–14^

The European SENTINA trial enrolled patients with node positive disease based on clinical examination alone (only 25% had a fine-needle aspirate of an axillary lymph node) and required patients to have a negative axilla sonographically after completion of NAC. Although overall, SLN surgery had a FNR of 14.2% (32/226); when limited to patients with greater than one SLN resected, the FNR was 9.6% (15/156). The ACOSOG Z1071 trial enrolled 756 patients with clinical node positive disease proven by percutaneous needle biopsy. Of the 525 eligible patients with clinical N1 disease, where 2 or two more SLNs were resected, the FNR of SLN surgery was 12.6% (39/310). This study further showed that in the cases where both radioactive colloid and blue dye were utilized to identify the SLNs, the FNR was reduced to 10.8% (27/251). When immunohistochemistry was performed on the SLNs, the FNR was 8.7%. In the 170 cases where a clip was placed in the positive lymph node at diagnosis, a subset analysis showed that with resection of the clipped node at the time of SLN surgery, the FNR was 6.8%.^15^ The Canadian SN-FNAC trial enrolled a total of 153 patients and reported a FNR of 13.3% when limiting node positive disease to nodal metastases >0.2 mm; it was reduced to 8.4% when cases with isolated tumor cells [N0(i+)] were assessed as node positive. The study also showed that the FNR was lower when two or more SLNs were resected (4.9%) and when dual tracer was utilized for SLN identification (5.2%).

Subsequently, based on the clipped node data from the Z1071 trial, the group at MD Anderson Cancer Center adjusted their practice to preoperatively localize and ensure resection of the clipped node at the time of SLN surgery. They describe this as ‘targeted axillary dissection’ and reported a FNR of 2.4% with the removal of the SLNs and the localized clipped node.^16^ The Netherlands Cancer Institute has utilized a technique called “MARI—marking the axillary lymph node with a radioactive seed” in which the radioactive seed is placed in the lymph node at the time of initial percutaneous biopsy prior to chemotherapy and then this radioactive seed localized lymph node only is resected at the time of surgery without SLN surgery. They reported a FNR of 7% (65/70).^17^ Alternative methods to mark the positive node at initial biopsy have also been explored including tattooing the node with ink or charcoal.^18,19^

## Why a need for surgical standards for axillary management in clinical trials?

**Fig. 1:**
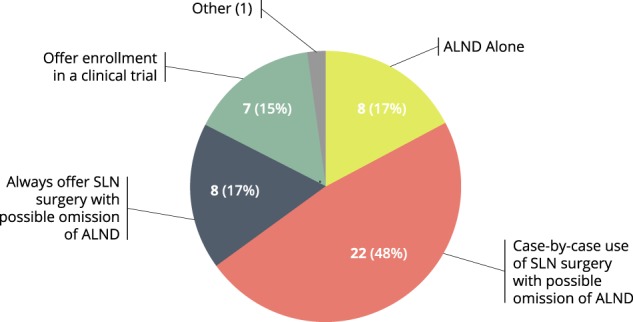
Current methods of management of axilla at the time of surgery in node positive patients who undergo NAC, as reported by participating surgeons surveyed in the I-SPY2 trial

With these advances, the surgical management of the axilla in patients treated with NAC is increasingly varied between practices. In the multicenter clinical trial setting, this can be problematic, introducing an additional source of variance in outcome assessment. In clinical trials with pCR as the primary endpoint evaluating therapies for potential FDA approval, accurate assessment of the axillary nodes after neoadjuvant chemotherapy is crucial. The I-SPY2 trial program therefore set out to evaluate the axillary surgery practices of surgeons at its current and prospective clinical trial sites. A survey was distributed to the surgeons at fourteen participating I-SPY2 sites and fifty-two surgeons at prospective national and international I-SPY3 sites. A total of 46 responses were collected. Survey results demonstrated a wide variation in surgical approaches to the axilla for clinically node positive patients treated with neoadjuvant chemotherapy who are clinically node negative at the time of surgery (Fig. 1).

Routine SLN surgery with the possibility of ALND depending on pathology was reported by 17.4% (*N* = 8), while most surgeons (*N* = 22, 47.9%) report offering SLN surgery with the possibility of omitting ALND on a case-by-case or selective basis. Routine ALND was recommended by 17.4% (*N* = 8). Enrollment in a clinical trial on axillary management was choosen by seven surgeons (15%).

Additionally, when surveyed regarding their approach to SLN surgery, surgeons reported differing protocols for performing SLN surgery in the setting of a patient who was clinically node positive treated with NAC. In the 39 surgeons who reported performing SLN surgery in this setting, most (*N* = 21, 53.8%) reported their protocol for identifying the SLNs utilized dual mapping agents with removal of the previous clipped node(s) and removal of 2–3 nodes. Other methods utilized included dual mapping agents with or without removal of previously clipped nodes with a minimum of 2–3 nodes (*N* = 10, 25.6%), single agent mapping with removal of the clipped node(s) with a minimum of 2–3 lymph nodes (*N* = 7, 17.9%), and single agent mapping with or without the removal of the clipped nodes with a minimum of 2–3 nodes (*N* = 1, 2.6%).

Therefore, in the setting of clinical trials evaluating response to systemic therapy, given the importance of accurate staging of the axillary lymph nodes along with optimizing the clinical management of patient care and avoiding over-treatment and invasive axillary surgery, there is a need to standardize the optimal surgical management of the axilla for patients treated with neoadjuvant chemotherapy on prospective clinical trials evaluating pCR as a primary endpoint. The multidisciplinary team from the I-SPY2 clinical trial leadership along with their principal investigators including surgeons, medical oncologist, and pathologist across sites have developed a recommended minimum standard, as follows, for surgical management of the axilla in patients with clinically node positive disease treated with neoadjuvant chemotherapy. The goal of these guidelines is to set a standard that surgeons adhere to when treating patients on a clinical trial with a pCR primary endpoint and can also be used to inform management outside of a clinical trial.

## I-SPY2 recommended standards

**Fig. 2:**
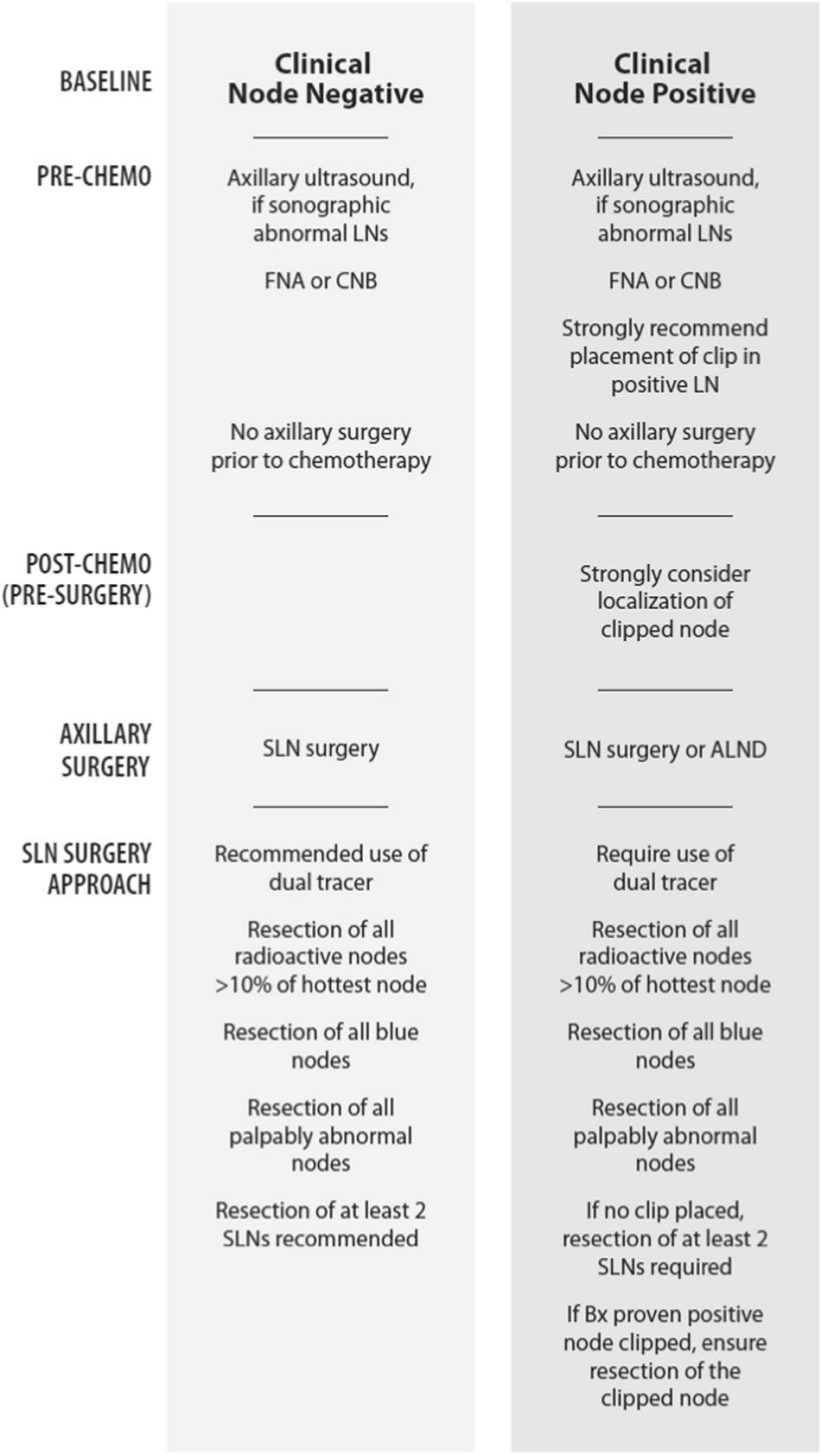
Recommended standards for axillary management in clinical trials of neoadjuvant therapy for breast cancer where pCR is the primary endpoint, for clinical node negative and clinical node positive disease at time of diagnosis

The following protocols have been adopted as standard minimum procedure by the I-SPY2 trial (NCT01042379), for management of axilla in patients with node positive disease (see Fig. 2).

## Initial diagnosis (baseline)

At initial diagnosis with breast cancer, prior to chemotherapy, placement of a clip at the primary tumor site at the time of diagnosis is standard for all patients treated with neoadjuvant systemic therapy. Additionally, evaluation should include documentation of the clinical nodal status by physical examination and axillary ultrasound. Cases with sonographically abnormal lymph nodes (cortex > 3 mm) should undergo percutaneous needle biopsy (fine needle aspiration or core needle biopsy) of the most abnormal lymph node, for cytologic or histologic evaluation for the presence of metastatic disease. Placement of a clip in the biopsy-proven positive node is strongly recommended, to aid identification of this node at surgery after completion of chemotherapy. Clip placement can be performed at the time of fine needle aspiration biopsy or core needle biopsy of the lymph node or at a subsequent time point if needed. If multiple nodes are biopsied, the most abnormal appearing node should be clipped. Placement of the clip in the hilum of the lymph node can avoid potential issues with clip migration during response to chemotherapy. Surgical staging of the axilla must occur after neoadjuvant chemotherapy. Axillary surgery before neoadjuvant chemotherapy loses the ability to assess response to therapy and to document the presence or absence of residual disease, therefore in trials with pCR as the primary endpoint all axillary surgery must occur after completion of neoadjuvant chemotherapy.

## Surgical management after neoadjuvant therapy

Surgery for resection of the breast primary and staging of the axillary nodes should be performed no earlier than 3 weeks after last dose of cytotoxic chemotherapy and no later than 12 weeks after the last dose of cytotoxic chemotherapy. All patients must undergo resection of the site of primary disease in the breast by either breast conservation or mastectomy. In cases with complete imaging response, surgical resection is still required to assess for residual disease at the site of the index tumor as this is a critical component of response assessment for evaluation of new drugs. The resection is guided by the residual tumor seen on imaging in relation to the clip placed at diagnosis. If the tumor is no longer present on imaging, the entire surgical bed does not need to be removed, and thus placement of multiple clips to map out the extent of a single tumor at presentation is discouraged. The area of the tumor must be sampled to confirm pCR, and the clip serves as guidance for such a resection. When the original tumor is diffuse and hormone receptor positive, it is more likely that scattered disease may be present over the area of the tumor bed,^20^ and this may impact the approach to surgical resection.

The American Joint Committee on Cancer ypT catgeory is based on the size of the largest contiguous focus of residual viable tumor, excluding areas of fibrotic tumor bed. A more comprehensive method of pathological evaluation of response after neoadjuvant chemotherapy is the residual cancer burden (RCB), which accounts for intervening fibrosis by incorporating the largest two-dimensional area of extent of residual invasive cancer and the percent of that area containing cancer cells, percentage of cancer that is in situ disease, and the extent of nodal involvement by the number of involved lymph nodes, and size of largest diameter of metastasis in the lymph nodes.^21^ I-SPY2 uses the methodology of mapping the histopathologic sections to the macroscopic findings as its standard procedure to accurately determine the extent of residual disease for ypStage and RCB, to maintain standardized pathology procedures across clinical sites and minimize the chance of missing residual disease.

## Axillary surgical management after neoadjuvant therapy

### Clinically node negative disease

Patients with no abnormal lymph nodes by examination and those whose needle biopsy is negative are classified as clinically node negative (cN0). In these cases, axillary surgical staging after neoadjuvant chemotherapy with SLN surgery is strongly recommended. Axillary dissection is permitted in cases with failure to identify SLNs. Use of dual tracer mapping (a blue dye and a radiolabeled agent or magnetic tracer) is recommended and resection of at least two SLNs is recommended. Careful evaluation of the axilla should be performed, including palpation and resection of all blue nodes and all radioactive nodes with counts >10% of the hottest node. All palpable nodes that are suspicious should also be resected.

### Clinically node positive disease at diagnosis

Those patients with positive needle biopsy at diagnosis (prior to chemotherapy) are classified as clinically node positive [cN1-3(f)]. Additionally, in patients with abnormal nodes by palpation and those with a positive PET CT or equivalent staging exam, percutaneous needle biopsy for histologic/cytologic evaluation is strongly recommended, with placement of a clip in the positive node. Although PET-positive axillary nodes are often assumed to be involved, biopsy and placement of a clip in those nodes is recommended as it enables targeting of the node for removal and evaluation after neoadjuvant chemotherapy. cN1 disease includes patients without matted nodes, even if several/multiple nodes appear abnormal on ultrasound or MRI. cN2 requires clinically fixed or matted nodes on examination or clinically or imaging detected internal mammary nodal involvement.

After completion of neoadjuvant chemotherapy, surgical management may include either SLN surgery or ALND. If SLN surgery is performed, dual tracer mapping is required (with the use of blue agent as well as a radiolabeled agent or MagTrace) along with careful evaluation of the axilla, including palpation, resection of radioactive nodes with a count greater than 10% of the hottest node, and resection of all blue nodes. All palpable nodes that are suspicious should also be resected. In cases where a clip was placed in the positive node at diagnosis, the clipped node should be resected, and this can be achieved by any of several different methods. The clipped node may be identified as one of the SLNs (this will occur in approximately 75% of cases). Intraoperative palpation or ultrasound can be used to help find the clipped node, or alternatively, preoperative localization of the clipped node with a wire or radioactive or localizable seed or tattoo to ensure its identification can be performed. Direct visualization of the clip, specimen radiography or intraoperative pathologic assessment is necessary to confirm that the clipped node has been resected and should be documented. Pathology is recommended to report the histologic findings of the clipped node specifically in their report. If the clipped node is not resected, then ALND is strongly recommended. In cases with node positive disease at presentation, where a clip was not placed in the positive node, pathology is recommended to comment on the presence or absence of biopsy site changes in the SLNs. In the absence of a clip, a minimum of two SLNs are required to be resected after completion of neoadjuvant chemotherapy and if this is not achieved ALND is required.

### Positive SLN

In all patients (cN0 or cN+) with a positive SLN after neoadjuvant chemotherapy, the standard recommendation is ALND. However, the additional management of the axilla (ALND and/or axillary radiation) is at the discretion of the treating multidisciplinary team. For clinical trials such as I-SPY2, where the primary endpoint is pCR versus no pCR, in cases where any of the SLNs are positive, the patient will be classified as having RCB class 2 (RCB 2) disease at a minimum. If the clinical trial endpoint requires the distinction between RCB 2 and 3, or if the number of nodes involved will determine the radiation treatment fields, then ALND would be required for complete assessment of number of positive nodes.

## Conclusion

Advances in the surgical management of the axilla have led to changes in practice and more judicious use of ALND that may minimize morbidity from surgery. From the viewpoint of drug development, assessment of response to neoadjuvant chemotherapy remains paramount, and appropriate assessment of residual disease—the primary endpoint—is critical. Therefore minimizing the FNR of axillary surgery to ensure more consistency in clinical trial data, without mandating ALND for all patients is necessary. The key elements to improve the accuracy of the assessment include proper staging and identification of nodal involvement at diagnosis, and appropriately targeted management of the axilla at the time of surgical resection. The I-SPY2 trial team revised their protocol, establishing requirements for surgeons treating patients on the trial as outlined above, to maximize value of axillary assessment, minimize surgical complications, enable modern axillary management and prevent drop off in enrollment. At the same time, standardizing the protocol helps maintain fidelity of the primary endpoint and generate appropriate data to continue to advance drug development. In the era of trials that promote a precision medicine approach, we have made the appropriate improvements to surgical management of the axilla and allow the standards and treatment to evolve accordingly. These surgical standards would be appropriate for neoadjuvant clinical trials with pCR as endpoint and for clinical practice.

### Data availability statement

The data sets generated during and/or analyzed during the current study are available from the corresponding author on reasonable request.
