# Author Correction: Surgical Standards for Management of the Axilla in Breast Cancer Clinical Trials with Pathological Complete Response Endpoint

**DOI:** 10.1038/s41523-018-0096-0

**Published:** 2019-01-02

**Authors:** Judy C. Boughey, Michael D. Alvarado, Rachael B. Lancaster, W. Fraser Symmans, Rita Mukhtar, Jasmine M. Wong, Cheryl A. Ewing, David A. Potter, Todd M. Tuttle, Tina J. Hieken, Jodi M. Carter, James W. Jakub, Henry G. Kaplan, Claire L. Buchanan, Nora T. Jaskowiak, Husain A. Sattar, Jeffrey Mueller, Rita Nanda, Claudine J. Isaacs, Paula R. Pohlmann, Filipa Lynce, Eleni A. Tousimis, Jay C. Zeck, M. Catherine Lee, Julie E. Lang, Paulette Mhawech-Fauceglia, Roshni Rao, Bret Taback, Constantine Goodellas, Margaret Chen, Kevin M. Kalinsky, Hanina Hibshoosh, Brigid Killelea, Tara Sanft, Gillian L. Hirst, Smita Asare, Jeffrey B. Matthews, Jane Perlmutter, Laura J. Esserman, I-SPY2 Investigators, A. Jo Chien, Andres Forero-Torres, Douglas Yee, Erin D. Ellis, Heather S. Han, Janice Lu, Anne M. Wallace, Kathy S. Albain, Anthony D. Elias, Amy S. Clark, Kathleen Kemmer

**Affiliations:** 1 Department of Surgery, Mayo Clinic, Rochester, MN USA; 2 UCSF Heller Diller Family Comprehensive Cancer Center, San Francisco, CA USA; 3 Department of Surgery, University of Alabama at Birmingham, Birmingham, AL USA; 4 Department of Pathology, MD Anderson Cancer Center, Houston, TX USA; 5 Department of Medicine, University of Minnesota, Minneapolis, MN USA; 6 Department of Surgery, University of Minnesota, Minneapolis, MN USA; 7 Department of Pathology, Mayo Clinic, Rochester, MN USA; 8 Swedish, Swedish Cancer Institute, Seattle, WA USA; 9 Department of Surgery, University of Chicago, Chicago, IL USA; 10 Department of Pathology, University of Chicago, Chicago, IL USA; 11 Department of Hematology and Oncology, University of Chicago, Chicago, IL USA; 12 Georgetown University Medical Center, Lombardi Cancer Center, Washington, USA; 13 Moffitt Cancer Center, Tampa, FL USA; 14 University of Southern California, Norris Comprehensive Cancer Center, Los Angeles, CA USA; 15 Columbia University Medical Center, New York, NY USA; 16 Loyola University Medical Centre, Maywood, IL USA; 17 Department of Surgery and Department of Medical Oncology, Yale University, New Haven, CT USA; 18 Quantum Leap Health Care Collaborative, San Francisco, CA USA; 19 Patient Representative, Ann Arbor, Michigan USA; 20 Department of Breast Oncology, Moffitt Cancer Center, Tampa, FL USA; 21 University of California, San Diego, La Jolla, CA USA; 22 University of Colorado Denver, Denver, CO USA; 23 University of Pennsylvania, Philadelphia, PA USA; 24 Oregon Health and Sciences, Portland, OR USA

**Correction to:** *npj Breast Cancer* **4**, 26 (2018) 10.1038/s41523-018-0074-6; Article Published online 17 August 2018

In the original version of the published article, Constantine Godellas was mistakenly omitted from the Author list and Author Contributions statement. Constantine Godellas has been added as the 29th Author, and is affiliated with Loyola University Medical Center, Maywood, IL, United States. The author contributions statement has been updated as follows: Author Contributions Statement: Judy C. Boughey conducted the review of current protocols, led the effort to establish new I-SPY standards and was the principal author of the manuscript. Laura Esserman co-led the review and standardization processes. Michael D. Alvarado, Rachael B. Lancaster, Fraser Symmans, W, Rita Mukhtar, Jasmine Wong, Cheryl Ewing, David Potter, Todd Tuttle, Tina Hieken, Jodi Carter, James Jakub, Henry Kaplan, Claire Buchanan, Nora Jaskowiak, Husain Sattar, Jeffrey Mueller, Rita Nanda, Claudine Isaacs, Paula Pohlmann, Filipa Lynce, Eleni Tousimis, Jay Zeck, M. Catherine Lee, Julie Lang, Paulette Mhawech-Fauceglia, Roshni Rao, Bret Taback, Margaret Chen, Kevin Kalinsky, Hanina Hibshoosh, Brigid Killelea, Constantine Godellas, and Tara Sanft provided medical and scientific expertise/opinion towards the development of I-SPY standards. Jane Perlmutter provided the patient advocate’s perspective in these discussions. All I-SPY2 trial investigators participated in the review of current standards, had the opportunity to participate in and comment on proposed standards and the final manuscript. Gill Hirst and Smita Asare provided expertise on, conducted and analyzed surveys of I-SPY trial sites. Jeffrey B. Matthews provided significant input and editing throughout the manuscript development process. All authors reviewed manuscript drafts and signed off on the final manuscript. This has been corrected in the HTML and PDF version of the article.

